# Staurosporine induces different cell death forms in cultured rat astrocytes

**DOI:** 10.2478/v10019-012-0036-9

**Published:** 2012-11-09

**Authors:** Janez Simenc, Metoda Lipnik-Stangelj

**Affiliations:** 1 University of Ljubljana, Faculty of Medicine, Ljubljana, Slovenia; 2 University of Maribor, Faculty of Medicine, Maribor, Slovenia

**Keywords:** astrocytes, staurosporine, necroptosis, apoptosis, reactive oxygen species, flow cytometry

## Abstract

**Background:**

Astroglial cells are frequently involved in malignant transformation. Besides apoptosis, necroptosis, a different form of regulated cell death, seems to be related with glioblastoma genesis, proliferation, angiogenesis and invasion. In the present work we elucidated mechanisms of necroptosis in cultured astrocytes, and compared them with apoptosis, caused by staurosporine.

**Materials and methods:**

Cultured rat cortical astrocytes were used for a cell death studies. Cell death was induced by different concentrations of staurosporine, and modified by inhibitors of apoptosis (z-vad-fmk) and necroptosis (nec-1). Different forms of a cell death were detected using flow cytometry.

**Results:**

We showed that staurosporine, depending on concentration, induces both, apoptosis as well as necroptosis. Treatment with 10^−7^ M staurosporine increased apoptosis of astrocytes after the regeneration in a staurosporine free medium. When caspases were inhibited, apoptosis was attenuated, while necroptosis was slightly increased. Treatment with 10^−6^ M staurosporine induced necroptosis that occurred after the regeneration of astrocytes in a staurosporine free medium, as well as without regeneration period. Necroptosis was significantly attenuated by nec-1 which inhibits RIP1 kinase. On the other hand, the inhibition of caspases had no effect on necroptosis. Furthermore, staurosporine activated RIP1 kinase increased the production of reactive oxygen species, while an antioxidant BHA significantly attenuated necroptosis.

**Conclusion:**

Staurosporine can induce apoptosis and/or necroptosis in cultured astrocytes via different signalling pathways. Distinction between different forms of cell death is crucial in the studies of therapy-induced necroptosis.

## Introduction

In the central nerve system (CNS), astroglial cells are frequently involved in malignant transformation. It is believed that dysfunction of apoptosis underlies glioblastoma genesis, proliferation and resistance to chemotherapy and radiotherapy. Besides, necrosis seems to be related with glioblastoma proliferation, angiogenesis and invasion. Induction of apoptosis has not made sufficient achievements in the treatment of glioblastoma, mainly because the tumour cells are often resistant to apoptosis. Better result in this case may be achieved by modulating the necroptosis, thus circumvent the apoptosis resistance.^1^ The knowledge of molecular pathways, involved in different forms of cell death is therefore crucial.

Apoptosis is a well-studied form of programmed cell death, with morphological characteristics such as cell shrinkage, fragmentation of cellular organelles and nucleus. In early apoptosis plasma membrane remains largely intact however it redistributes phosphatydilserine from the cytoplasmatic to the outer surface prior to any morphological changes. In late apoptosis, plasma membrane often becomes permeable, causing secondary necrosis.^2^,^3^ Described changes are mostly due to the activated intracellular cysteine-proteases caspases.^4^ In contrast, primary necrosis is morphologically characterized by swelling of the cytoplasm and cell organelles, followed by early plasma membrane ruptures. The release of intra-cellular content outside of the cell may induce an inflammatory response and additional cell loss. Contrary to apoptosis, primary necrosis does not involve caspases.^3^ For long time, primary necrosis has only been considered as an unregulated, passive cell death caused by the devastating stress. However, at least a part of necrotic cells may die by necroptosis, a highly regulated form of primary necrosis.^5^

Apoptosis and necroptosis are rather interconnected and not entirely separated events. In different susceptible cell lines, Fas ligand or TNF-alpha, which normally activates apoptosis through the death receptors, can induce necroptosis when caspases are inhibited or deficient.^6^ While the mechanisms of apoptosis are well known, necroptosis is not fully understood. One critical molecular regulator, mediating different cellular responses upon activation through the death receptors, is a multifunctional receptor interacting protein (RIP) which is essential for the activation of necroptosis, when caspases are inhibited.^6^ Recent reports revealed several details of necroptotic death signalling pathway and have confirmed a critical role of RIP1 kinase in necroptosis.^7^,^8^

In the central nerve system, astroglial cells in the brain may die by apoptosis or necroptosis in different pathologies.^9^–^11^ Distinction of the mechanisms of astrocytes apoptosis and necroptosis is necessary to improve our understanding of pathophysiology of neurological disorders, cancer or trauma and provide new therapy opportunities. Astroglial cell cultures have proven an appropriate *in vitro* model for studying cellular and molecular functions of astrocytes, including cell death.^12^ Recently we have reported, that staurosporine is able to induce necroptosis in cultured astrocytes.^13^ So far, the signalling pathways of staurosporine induced necroptosis have not been elucidated. Therefore in the present study we clarified the molecular mechanisms of necroptosis, induced by staurosporine, and compared them with apoptosis. We showed that necroptosis is caspases independent and occurs through the activation of RIP1 kinase. Furthermore, we demonstrated that the reactive oxygen species (ROS) production is increased through RIP1 kinase activity, while increased ROS is associated with necroptosis.

## Materials and methods

### Materials

L-15 Leibowitz medium, foetal bovine serum (FBS), Dulbecco’s modified Eagle medium and Ham’s nutrient mixture F-12 (DMEM/F12), Penicillin (10,000 IU/ml), Streptomycin (10,000 mg/ml; P/S), Dulbecco’s phosphate buffered saline (PBS) were purchased from Gibco BRL, Life Technologies, Scotland. Staurosporine, 2–7-dichlorodihydrofluorescin diacetate (DCFH-DA) probe, Necrostatin-1(nec-1), and Butylated hydroxyanisole (BHA) were obtained from Sigma Chemicals, USA. The pan-caspase inhibitor (z-vad-fmk) was purchased from R&D systems, USA. Petri plates were purchased from Nunc, Germany, and tissue culture flasks were obtained from TPP, Switzerland. Annexin V-fluorescin isothiocyanate (Annexin V-FITC) and 7-Aminoactinomycin D (7-AAD) staining kit for a flow cytometry was obtained from Beckman-Coulter, USA. All flow cytometry experiments were carried out on the Quanta SC MPL flow cytometer (Beckman Coulter, USA).

### Animals

New-born Wistar rats (1–2 days old) were obtained from our own breeding colony. All animal studies were approved by the Veterinary Authority of the Republic of Slovenia (License number: 34401-80/2008/4), and performed in accordance with the EU Directive 2010/63/EU and the European Convention for the protection of vertebrate animals used for experimental and other scientific purposes (ETS 123).

### Cell cultures

Cultures of rat cortical astrocytes were prepared from the brain of new-born rats in DMEM/F12 (1:1), 10% FBS, 1% Penicillin-Streptomycin culture medium as described previously.^13^ Cells were grown at 37°C in a humidified environment containing 10% CO2 until they became confluent. When they reached confluence, the cells were used for the treatment.

### Induction of cell death and production of ROS by staurosporine

The astrocytes were treated with 10^−7^ M staurosporine for 6 hours to induce apoptosis. After the treatment, the cells were allowed to regenerate for 22 hours in a staurosporine free medium, or were analysed without regeneration. Similarly, the astrocytes were treated with 10^−6^ M staurosporine for 3 hours to induce the production of ROS and/or necroptosis. After the treatment, the cells were allowed to regenerate for 22 hours in a staurosporine free medium, or were analysed without regeneration. The cells were trypsinized and stained for an analysis with a flow cytometer. The control cells were not exposed to staurosporine.

### Attenuation of apoptosis

The astrocytes were pre-treated with z-vad-fmk, an irreversible pan-caspase inhibitor, at 4 x 10^−5^ M concentration, for one hour. Then 10^−7^ M staurosporine was added into the culture medium, and the cells were incubated for an additional 6 hours. During 22 hours regeneration, the cells were incubated in z-vad-fmk containing medium without staurosporine. The cells were also exposed only to 4 x 10^−5^ M z-vad-fmk for 24 hours.

### Attenuation of necroptosis and ROS production

The astrocytes were pre-treated with 10^−4^ M nec-1, a specific RIP1 kinase inhibitor, or with 4 x 10^−5^ M z-vad-fmk, one hour before 10^−6^ M staurosporine was added. Then the cells were incubated for an additional 3 hours. For the regeneration, the cells were incubated in the presence of nec-1 or z-vadfmk for 22 hours. The cells were exposed only to 10^−4^ M nec-1 for 24 hours as well. For the attenuation of ROS production, the cells were pre-treated with 10^−4^ M nec-1, one hour before 10^−6^ M staurosporine was added. Then the cells were incubated for additional 3 hours. For the regeneration, the cells were incubated in the presence of nec-1 for 22 hours. Additionally, to attenuate the ROS associated necroptosis, the cells were treated one hour with 10^−4^ M BHA, a ROS scavenger, before 10^−6^ M staurosporine was added. Then the cells were incubated for an additional 3 hours. For the regeneration, the cells were incubated in the presence of BHA for 22 hours. The cells were exposed only to 10^−4^ M BHA for 24 hours as well.

### Flow cytometric analysis of a cell death and ROS production

The cells were stained simultaneously with Annexin V-FITC and 7-AAD dye according to the modified manufacturer’s instructions (for details see Šimenc&Lipnik-Štangelj^13^). Data acquisition was carried out using a flow cytometer. The differentiation of early apoptotic, secondary necrotic, necroptotic and viable cells was made according to their phenotype: Annexin V^+^/7-AAD^−^ were considered early apoptotic, Annexin V^−^/7-AAD^+^, necroptotic, Annexin V^+^/7-AAD^+^ secondary necrotic, and Annexin V^−^/7-AAD^−^ viable cells. In each sample, 10.000 cells were analysed.

For the detection of ROS production, the cells were prepared according to the modified protocol.^14^

### Statistical analyses

Statistical analyses were made with SPSS 19 software (SPSS, Inc, USA). For each treatment and controls, ten samples from two independent groups of animals were analysed. For inhibitors toxicity (z-vad-fmk or nec-1), five samples were analysed. In the cell death experiments, data (means ± SEM) were expressed as the percentage of cell death. For statistical comparisons, only the proportions of early apoptotic and necroptotic cells were considered. For the ROS production, data (means ± SEM) were expressed as the percentage of DCF fluorescence. The differences between various groups were examined for a significance using the non-parametric Mann-Whitney U test. In all cases a p value of < 0.01 was considered statistically significant.

## Results

### Determination of apoptosis and necroptosis

**FIGURE 1.**
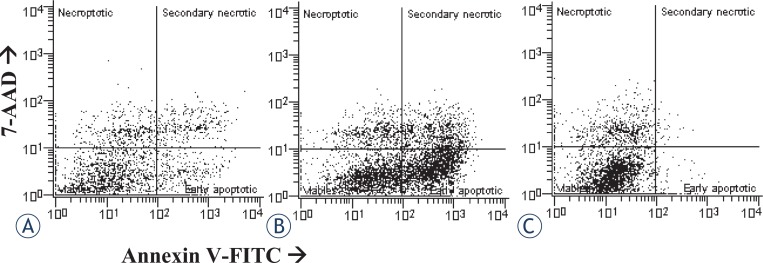
Examples of two-parameter flow cytometry dot plots showing simultaneous binding of Annexin V and 7-AAD uptake by cultured rat astrocytes after the induction of apoptosis. For each treatment, ten samples from two independent groups of animals were analysed. (A) Cells were not exposed to staurosporine (controls). (B) Cells were exposed to 10^−7^ M staurosporine for 6 hours, and regenerated for 22 hours in a staurosporine free medium. (C) Cells were exposed to 10^−7^ M staurosporine for 6 hours, and analysed without regeneration.

**FIGURE 2.**
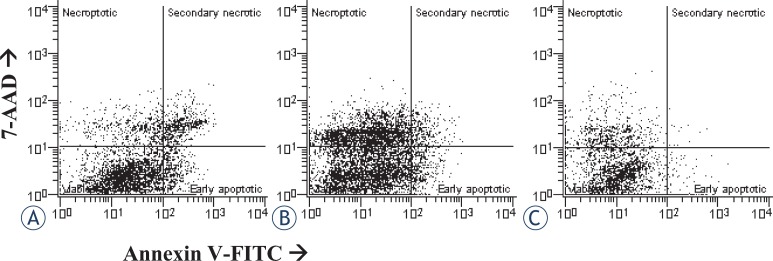
Examples of two-parameter flow cytometry dot plots, showing the simultaneous binding of Annexin V and 7-AAD uptake by cultured rat astrocytes after the induction of necroptosis. For each treatment, ten samples from two independent groups of animals were analysed. (A) Cells were not exposed to staurosporine (controls). (B) Cells were exposed to 10^−6^ M staurosporine for 3 hours, and regenerated for 22 hours in a staurosporine free medium. (C) Cells were exposed to 10^−6^ M staurosporine for 3 hours, and analysed without regeneration.

Apoptosis and necroptosis were detected by the flow cytometry dot plots (Figures 1, 2). The proportions of apoptotic and necroptotic cells were simultaneously detected with the binding of Annexin V-FITC and uptake of 7-AAD dye. The binding of Annexin V is considered independent of a cell death stimulus and specific marker of apoptosis. It precedes the loss of ability to exclude viability dyes, membrane ruptures, or occurrence of any morphological changes associated with apoptosis.^15^,^16^ On the other hand, the uptake of 7-AAD dye is a specific marker of necroptosis.^17^ However, it has been reported for the Jurkat cells with necrotic morphology, that the binding of Annexin V^+^ precedes the formation of membrane ruptures and necrosis.^6^ This observation suggests that secondary necrotic cells (Annexin V^+^/7-AAD^+^) may not necessarily die by apoptosis. Therefore, to avoid potential bias, secondary necrotic cells were omitted from the analyses, and only early apoptotic cells (Annexin-V^+^ / 7-aad^−^) were considered apoptotic. Accordingly, only the cells with Annexin V^−^/7-AAD^+^ phenotype were considered as necroptotic cells, while secondary necrotic cells were omitted from the analyses.

### Induction and attenuation of apoptosis and necroptosis

**FIGURE 3.**
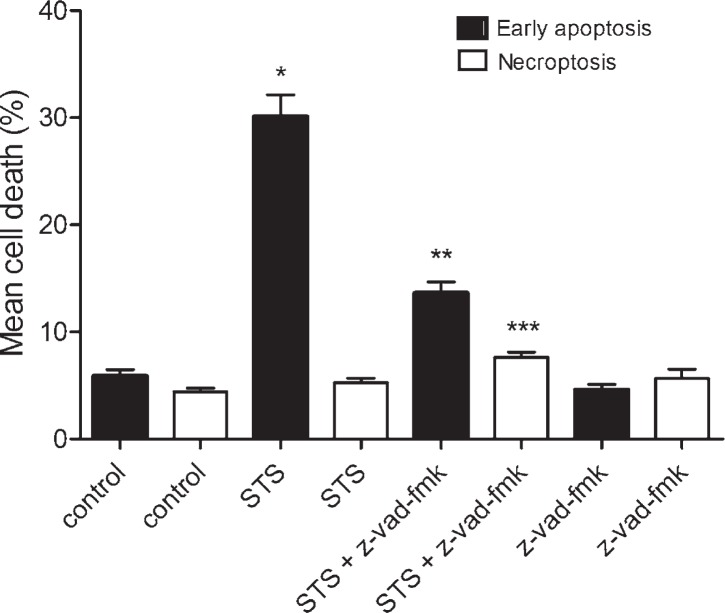
Percentages of a cell death in cultured rat astrocytes after the exposure to 10^−7^ M staurosporine. Early apoptosis and necroptosis were determined by the binding of Annexin V-FITC and 7-AAD uptake, using a flow cytometry. Data are the means ± SEM of ten samples from two independent groups of animals. For the z-vad-fmk treatment, only five samples were analysed. (Control) Control cells were not exposed to staurosporine or z-vad-fmk. (STS) Cells were exposed to 10^−7^ M staurosporine for 6 hours, and regenerated for 22 hours in a staurosporine free medium. (STS+z-vad-fmk) Cells were exposed to staurosporine and 4 x 10^−5^ M z-vad-fmk for 6 hours, and regenerated for 22 hours in a staurosporine free medium with z-vad-fmk. (z-vad-fmk) Cells were exposed to 4 x 10^−5^ M z-vadfmk for 24 hours. Data were analysed using the non-parametric Mann-Whitney U test; * p < 0.00 *vs* Control-early apoptosis, ** p < 0.00 *vs* STS-early apoptosis and *** p = 0.002 *vs* STS-necroptosis indicate significance.

For the induction of apoptosis, the cells were exposed to 10^−7^ M of staurosporine for 6 hours, and regenerated for 22 hours in a staurosporine free medium. As shown in Figure 3, staurosporine increased early apoptosis approximately 5-fold in comparison to control cells, while necroptosis was not influenced. In order to confirm the induction of apoptosis, inhibition experiments with z-vadfmk were carried out. The results showed that inhibition of caspases by z-vad-fmk strongly reduced early apoptosis, induced by staurosporine. On the contrary, the number of necroptotic cells was slightly increased, suggesting that the cells were switched from one form of a cell death (apoptosis) to another (necroptosis) (Figure 3).

**FIGURE 4.**
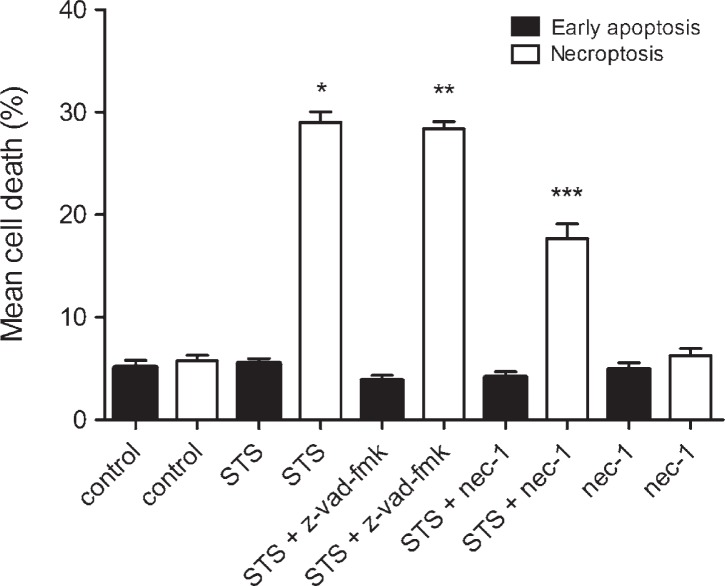
Percentages of a cell death in cultured rat astrocytes after the exposure to 10^−6^ M staurosporine and the regeneration period. Early apoptosis and necroptosis were determined by the binding of Annexin V-FITC and 7-AAD uptake, using a flow cytometry. Data are the means ± SEM of ten samples from two independent groups of animals. For the nec-1 treatment, only five saples were analysed. (Control) Control cells were not exposed to staurosporine or inhibitors. (STS) Cells were exposed to 10^−6^ M staurosporine for 3 hours, and regenerated for 22 hours in a staurosporine free medium. (STS+z-vad-fmk) Cells were exposed to staurosporine and 4 x 10^−5^ M z-vad-fmk for 3 hours, and regenerated for 22 hours in a staurosporine free medium with the z-vad-fmk. (STS+nec-1) Cells were exposed to staurosporine and 10^−4^ M nec-1 for 3 hours, and regenerated for 22 hours in a staurosporine free medium with nec-1. (nec-1) Cells were exposed to 10^−6^ M nec-1 for 24 hours. Data were analysed using the non-parametric Mann-Whitney U test; * p < 0.00 *vs* Control-necroptosis, ** p < 0.00 *vs* Control-necroptosis and *** p < 0.00 *vs* STS-necroptosis indicate significance.

To induce necroptosis, cell cultures were exposed to 10^−6^ M staurosporine for 3 hours, and regenerated as described previously. The results showed that a higher concentration of staurosporine increased the extent of necroptosis (staurosporine 29.01 ± 1.03% *vs* control cells 5.76 ± 0.54%), whereas early apoptosis was not affected. To confirm the RIP1 kinase involvement in the transduction of the signal, we inhibited necroptosis with nec-1. The results showed that nec-1 reduced staurosporine induced necroptosis to half, while early apoptosis remained unaffected. Furthermore, z-vad-fmk did not influence necroptosis which indicating that necroptosis, induced by staurosporine, was caspases independent (Figure 4).

### Induction and attenuation of ROS production and ROS associated necroptosis

**FIGURE 5.**
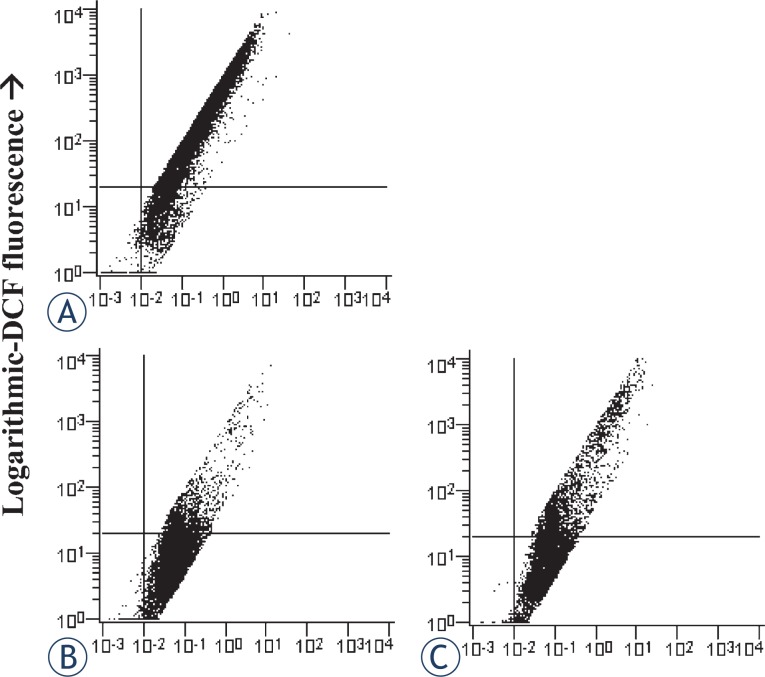
Detection of ROS production in cultured rat astrocytes by a flow cytometry. Examples of the forward scatter dot plots of the logarithmic versus linear amplified DCF fluorescence. Percentages of the cells from lower right quadrants were compared for the ROS production. For each treatment, ten samples from two independent groups of animals were analysed. (A) Cells were not exposed to staurosporine. (B) Cells were exposed to 10^−6^ M staurosporine for 3 hours, and regenerated for 22 hours in a staurosporine free medium. (C) Cells were exposed to 10^−6^ M staurosporine and 10^−4^ M nec-1 for 3 hours, and regenerated for 22 hours in a staurosporine free medium with nec-1.

**FIGURE 6.**
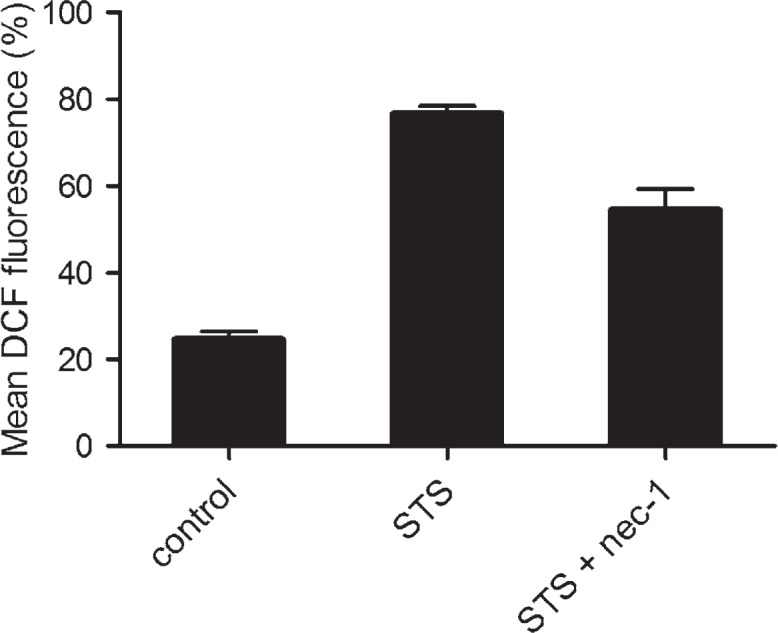
Production of ROS, detected as the percentage of DCF fluorescent cells. Data are the means ± SEM of ten samples from two independent groups of animals. (Control) Cells were not exposed to staurosporine. (STS) Cells were exposed to 10^−6^ M staurosporine for 3 hours, and regenerated for 22 hours in a staurosporine free medium. (STS+nec-1) Cells were exposed to 10^−6^ M staurosporine and 10^−4^ M nec-1, for 3 hours, and regenerated for 22 hours in a staurosporine free medium with nec-1. Data were analysed using the non-parametric Mann-Whitney U test; * p < 0.00 *vs* Control, ** p = 0.001 *vs* STS indicate significance.

In the cultures, exposed to 10^−6^ M staurosporine, the ROS accumulation was detected by DCF fluorescence. Linear amplification of the signal was observed in the staurosporine treated cells. Analyses were made by the logarithmically versus linear amplified DCF fluorescence cytometry dot plots (Figure 5). As shown in Figure 6, staurosporine increased the ROS production approx. 3-fold in comparison to control cells. Nec-1 reduced the ROS production, induced by staurosporine, to half.

**FIGURE 7.**
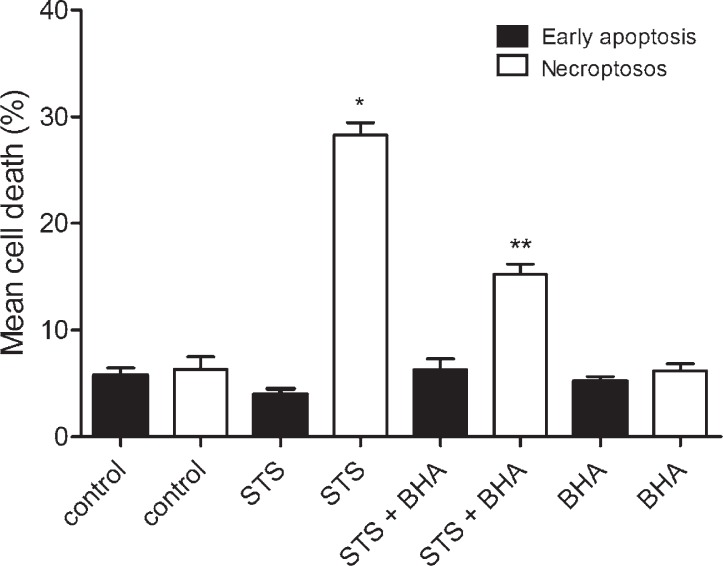
Percentages of a cell death in cultured rat astrocytes after the exposure to 10^−6^ M staurosporine and the regeneration period. Early apoptosis and necroptosis were determined by the binding of Annexin V-FITC and 7-AAD uptake, using a flow cytometry. Data are the means ± SEM of ten samples from two independent groups of animals. For the BHA treatment, only five samples were analysed. (Control) Control cells were not exposed to staurosporine or inhibitor. (STS) Cells were exposed to 10^−6^ M staurosporine for 3 hours, and regenerated for 22 hours in a staurosporine free medium. (STS+BHA) Cells were exposed to staurosporine and 10^−4^ M BHA for 3 hours, and regenerated for 22 hours in a staurosporine free medium with the BHA. (BHA) Cells were exposed to 10^−4^ M BHA for 24 hours. Data were analysed using the non-parametric Mann-Whitney U test; * p < 0.00 *vs* CON-necroptosis, ** p < 0.00 *vs* STS-necroptosis indicate significance.

In order to show that increased ROS is associated with necroptosis, the cells were treated with 10^−6^ M staurosporine in the presence of 10^−4^ M BHA. As shown in Figure 7, BHA strongly reduced necroptosis, while early apoptosis was not influenced.

### Induction of a cell death without regeneration

**FIGURE 8.**
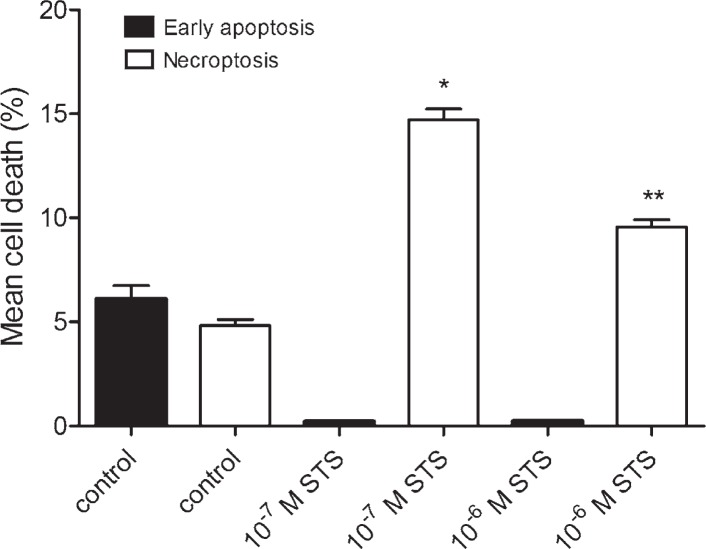
Percentages of a cell death in cultured rat astrocytes, induced by staurosporine, without the regeneration period. Cells were exposed either to 10^−7^ M staurosporine for 6 hours, or 10^−6^ M staurosporine for 3 hours, and analysed immediately. Early apoptosis and necroptosis were determined by the binding of Annexin V-FITC and 7-AAD uptake, using a flow cytometry. Data are the means ± SEM of ten samples from two independent groups of animals. (Control) Control cells were not exposed to staurosporine. (10^−7^ M STS) Cells were exposed to 10^−7^ M staurosporine. (10^−6^ M STS) Cells were exposed to 10^−6^ M staurosporine. Data were analysed using the non-parametric Mann-Whitney U test; * p < 0.00 *vs* Control-necroptosis, ** p < 0.00 *vs* Control-necroptosis indicate significance.

To assess the impact of regeneration period on a cell death, the experiments without regeneration were carried out. As shown in Figure 8, in the cultures treated with 10^−7^ M staurosporine for 6 hours, early apoptosis was significantly decreased, while necroptosis was increased. Similarly, in the cultures, treated with 10^−6^ M staurosporine for 3 hours, early apoptosis was significantly decreased, while necroptosis was increased (Figure 8). These results indicated that regeneration of the cells is crucial for apoptosis, while it is not necessary for necroptosis.

## Discussion

Death is an important issue in cell biology. Cells may die as a part of normal development or tissue homeostasis. On the other hand, they may die when they are damaged or infected. Cell death failure may underlay tumour genesis and resistance to chemotherapy or radiotherapy.

There are various cell death forms, which are differentiated by several morphological and functional criteria.^3^ The knowledge of the cell death mechanisms and tumorigenic properties of the cells, and development of advanced therapies is therefore a key to successful treatment.^18^,^19^

In our study we showed that staurosporine, depending on concentration, induces at least two different forms of a regulated cell death. When cultured astrocytes were exposed to 10^−7^ M staurosporine, a significant proportion of early apoptotic cells was observed in comparison to the control cells, while necroptosis was not influenced (Figure 1A,B). To confirm the induction of apoptosis, inhibition experiments with z-vad-fmk, an irreversible pancaspase inhibitor, were carried out. In the cultures, co-treated with z-vad-fmk, apoptosis was significantly attenuated. Importantly, as shown in Figure 3, necroptosis was slightly, yet significantly increased as compared to necroptosis in the staurosporine treated or control cultures. This observation suggests that staurosporine induced apoptosis and necroptosis are rather interconnected events in cultured rat astrocytes. Our results are in agreement with previously described induction of necroptosis instead of apoptosis, through the death receptors signalling, if caspases were inhibited.^6^

Staurosporine is widely used as a potent inducer of apoptosis. The mechanisms of apoptosis induction are diverse and may depend on the cell type.^20^ In mouse acute lymphoid leukaemia cells L1210, various human melanoma cell types, and human breast carcinoma cells MCF-7 have been shown that staurosporine induces apoptosis through both, rapid caspases dependent, and slow, caspase independent pathway.^21^–^23^ In cultured rat astrocytes, the exposure to 10^−7^ M staurosporine for 3 hours has induced delayed, caspase-3 dependent apoptosis.^24^ Our study expands these observations, as in addition to apoptosis, we induced necroptosis as well (Figure 2B). As shown in Figure 4, in the cultures exposed to a higher concentration of staurosporine, necroptosis was increased, while apoptosis was not.

Necroptosis is induced through RIP1 kinase activity.^6^–^8^ In order to confirm this hypothesis, RIP1 kinase was inhibited by its specific inhibitor, nec-1. Indeed, in the samples, co-treated with nec-1, necroptosis was significantly attenuated, as depicted in Figure 4. Necroptosis has also been shown as caspases-independent cell death^5^ which is in accordance with our results, where z-vad-fmk had no attenuating effect on necroptosis (Figure 4). We clearly show that the mechanism of necroptosis in cultured rat astrocytes, induced by staurosporine, is RIP1 kinase dependent and caspases independent. Apparently, at a higher concentration of staurosporine caspases play no role and astrocytes dye by necroptosis. Similar results were obtained in mouse astrocytes, where necroptosis, induced by hemin, was shown RIP1 kinase dependent and caspases independent.^11^ Taken together; necroptosis is an important form of astrocyte death, regardless of an initial stimulus.

In several cell types the reactive oxygen species (ROS) production has shown to be RIP1 kinase dependent.^24^,^25^ Similarly, we found that ROS were increased in the staurosporine treated astrocytes through the RIP1 kinase activity (Figure 5), while the production of ROS was attenuated, when the cells were co-treated with nec-1 (Figure 6). Furthermore, as shown in Figure 7, necroptosis was significantly reduced, when cells were co-treated with staurosporine and an antioxidant BHA. These observations clearly indicate that increased ROS contributes to necroptosis in a staurosporine treated rat astrocytes. Similarly, necroptosis, associated with ROS, was observed in hemin treated mouse astrocytes.^11^ It is conceivable that increased ROS production play an important role in astrocyte necroptosis.

Notably, apoptosis and necroptosis were significantly increased after a regeneration period in a staurosporine free medium. We elucidated therefore, whether regeneration is absolutely necessary. The astrocytes were exposed to different concentrations of staurosporine, and analysed without regeneration (Figures 1C, 2C). On the contrary to the experiments with regeneration, almost no apoptosis was detected, regardless of treatment, while necroptosis was already increased at 10^−6^ M as well as at 10^−7^ M staurosporine exposure (Figure 8). Whereas the regeneration seems not to be necessary for necroptosis, it is crucial for apoptosis. This observation indicates that the occurrence of necroptosis is much faster than of apoptosis. The later observation is in agreement with the reports of delayed apoptosis induced by staurosporine in various cell types including astrocytes.^21^–^23^ The delay in apoptosis may reflect a high resistance of astrocytes to apoptosis, which is in line with previously reported resistance of human fetal astrocytes to apoptosis, induced via cell death receptors.^26^

Reduced expression of different death receptors is also one of the crucial mechanisms of the resistance of glioblastoma to apoptosis.^28^ It can occur via mutated p53 pathway, which is the most commonly mutated pathway in tumorigenesis, and is strongly connected to apoptosis and necroptosis as well.^28^,^29^ Furthermore, mutation of human rat sarcoma (RAS) genes is also frequently associated with glioblastoma tumorigenesis. RAS regulates downstream mitogen-activated protein kinase (MAPK) signalling pathway and aberrant expression or defects in the RAS/MAPK pathway causes abnormal cellular activities such as invasion and cell death. Both, p53 and MAPK signalling pathways are associated with RIP1 kinase activity and necroptosis, and could be therefore important targets for glioma therapy.

In conclusion, staurosporine is able to induce two different forms of a cell death, apoptosis and necroptosis, in cultured astrocytes. Necroptosis is RIP1 kinase dependent and involves ROS production. Both cell death forms were significantly increased after the regeneration period; however increased necroptosis was already detected without regeneration. It seems that cultured astrocytes are more prone to necroptosis than apoptosis. Whether this is a general property of cultured astrocytes or it is confined to staurosporine only, requires further studies. As the demise of astrocytes is still ill understood, revealing the forms of their death may improve our understanding of the pathophysiology of various neurological disorders, malignant transformation, brain infections or traumatic brain injury. Moreover, our results may be useful in the studies of therapy-induced necroptosis in malignant transform cells. In this respect, the study provides a simple *in vitro* approach for the induction and detection of apoptosis and necroptosis in astroglial cells.

## References

[1] Jiang YG, Peng Y, Koussougbo KS (2011). Necroptosis: a novel therapeutic target for glioblastoma. Med Hypotheses.

[2] Taylor RC, Cullen SP, Martin SJ (2008). Apoptosis: controlled demolition at the cellular level. Nat Rev Cell Mol Biol.

[3] Kroemer G, Galluzzi L, Vandenabeele P, Abrams J, Alnemri ES, Baehrecke EH (2009). Classification of cell death: recommendations of the Nomenclature Committee on Cell Death. Cell Death Differ.

[4] Kumar S (2007). Caspase function in programmed cell death. Cell Death Differ.

[5] Degterev A, Huang Z, Boyce M, Li Y, Jagtap P, Mizushima N (2005). Chemical inhibitor of nonapoptotic cell death with therapeutic potential for ischemic brain injury. Nat Chem Biol.

[6] Holle N, Zaru R, Micheau O, Thome M, Attinger A, Valitutti S (2000). Fas triggers an alternative, caspase-8-independent cell death pathway using the kinase RIP as effector molecule. Nat Immunol.

[7] Cho YS, Challa S, Moquin D, Genga R, Ray TD, Guildford M (2009). Phosphorylation driven assembly of the RIP1 RIP3 complex regulates programmed necrosis and virus induced inflammation. Cell.

[8] Zhang DW, Shao J, Lin J, Zhang N, Lu BJ, Lin SC (2009). RIP3, an energy metabolism regulator that switches TNF-induced cell death from apoptosis to necrosis. Science.

[9] Rollins S, Perkins E, Mandybur G, Zhang JH (2002). Oxyhemoglobin produces necrosis, not apoptosis in astrocytes. Brain Res.

[10] Takuma K, Baba A, Matsuda T (2004). Astrocyte apoptosis: implications for neuro-protection. Prog. Neurobiol.

[11] Laird MD, Wakade C, Alleyne CH, Dhandapani KM (2008). Hemin induced necroptosis involves glutation depetion in mouse astrocytes. Free Radic Biol Med.

[12] Falsig J, Latta M, Leist M (2004). Defined inflammatory states in astrocyte cultures: correlation with susceptibility towards CD95-driven apoptosis. J Neurochem.

[13] Šimenc J, Lipnik-Štangelj M (2012). Staurosporine induces apoptosis and necroptosis in cultured rat astrocytes. Drug Chem Toxicol.

[14] Eruslanov E, Kusmartsev S, Armstrong D (2010). Identification of ROS using oxidized DCFDA and flow cytometry. Advanced protocols in oxidative stress II, Methods in Molecular Biology.

[15] Martin SJ, Reutelingsperger CEM, McGahon AJ, Rader JA, van Schie RCAA, LaFace DM (1995). Early redistribution of plasma membrane phosphatidylserine is a general feature of apoptosis regardless of the initiating stimulus: inhibition by overexpression of Bcl-2 and Abl. J Exp Med.

[16] Vermes I, Haanen C, Steffens-Nakken H, Reutelingsperger C (1995). A novel assay for apoptosis. Flow cytometric detection of phosphatidylserine expression on early apoptotic cells using fluorescein labelled Annexin V. J Immunol Methods.

[17] Schmid I, Uittenbogaart CH, Keld B, Giorgi JV (1994). A rapid method for measuring apoptosis and dual-color immunofluorescence by single laser flow cytometry. J Immunol Methods.

[18] Todorovic V, Sersa G, Mlakar V, Glavac D, Cemazar M (2012). Assessment of the tumourigenic and metastatic properties of SK-MEL28 melanoma cells surviving electrochemotherapy with bleomycin. Radiol Oncol.

[19] Vranic A (2011). New developments in surgery of malignant gliomas. Radiol Oncol.

[20] Stepczynska A, Lauber K, Engels IH, Janssen O, Kabelitz D, Wesselborg S (2001). Staurosporine and conventional anticancer drugs induce overlapping, yet distinct pathways of apoptosis and caspase activation. Oncogene.

[21] Belmokhtar CA, Hillion J, Ségal-Bendirdjian E (2001). Staurosporine induces apoptosis through both caspase-dependent and caspase-independent mechanisms. Oncogene.

[22] Xue LY, Chiu SM, Oleinick NL (2003). Staurosporine-induced death of MCF-7 human breast cancer cells: a distinction between caspase-3 dependent steps of apoptosis and the critical lethal lesions. Exp Cell Res.

[23] Zhang XD, Gillespie SK, Hersey P (2004). Staurosporine induces apoptosis of melanoma by both caspase dependent and independent apoptotic pathways. Mol Cancer Ther.

[24] D’Alimonte I, Ballerini P, Nargi E, Buccella S, Giuliani P, Di Iorio P (2007). Staurosporine-induced apoptosis in astrocytes is prevented by A1 adenosine receptor activation. Neurosci Lett.

[25] Kim SY, Morgan MJ, Choksi S, Liu ZG (2007). TNF induced activation of the Nox 1 NADPH oxidase and its role in the induction of necrotic cell death. Mol Cell.

[26] Song JH, Bellail A, Tse MCL, Wee Yong V, Hao C (2006). Human astrocytes are resistant to Fas ligand and tumor necrosis factor-related apoptosis inducing ligand induced apoptosis. J Neurosci.

[27] Sharon Biton S, Ashkenazi A (2011). NEMO and RIP1 control cell fate in Response to extensive DNA damage via TNF-α feedforward signaling. Cell.

[28] Mao H, Lebrun DG, Yang J, Zhu VF, Li M (2012). Deregulated signaling pathways in glioblastoma multiforme: molecular mechanisms and therapeutic targets. Cancer Invest.

